# In situ visualization of glycoside hydrolase family 92 genes in marine flavobacteria

**DOI:** 10.1038/s43705-021-00082-4

**Published:** 2021-12-18

**Authors:** Laura E. Zeugner, Karen Krüger, Jimena Barrero-Canosa, Rudolf I. Amann, Bernhard M. Fuchs

**Affiliations:** 1 Max Planck Institute for Marine Microbiology, Bremen, Germany; 2 Technical University of Berlin, Institute of Environmental Technology, Environmental Microbiology, Berlin, Germany

**Keywords:** Biological techniques, Marine microbiology, Water microbiology, Applied microbiology

## Abstract

Gene clusters rich in carbohydrate-active enzymes within *Flavobacteriia* genera provide a competitiveness for their hosts to degrade diatom-derived polysaccharides. One such widely distributed polysaccharide is glucuronomannan, a main cell wall component of diatoms. A conserved gene cluster putatively degrading glucuronomannan was found previously among various flavobacterial taxa in marine metagenomes. Here, we aimed to visualize two glycoside hydrolase family 92 genes coding for α-mannosidases with fluorescently-labeled polynucleotide probes using direct-geneFISH. Reliable in situ localization of single-copy genes was achieved with an efficiency up to 74% not only in the flavobacterial strains *Polaribacter* Hel1_33_49 and *Formosa* Hel1_33_131 but also in planktonic samples from the North Sea. In combination with high-resolution microscopy, direct-geneFISH gave visual evidence of the contrasting lifestyles of closely related *Polaribacter* species in those samples and allowed for the determination of gene distribution among attached and free-living cells. We also detected highly similar GH92 genes in yet unidentified taxa by broadening probe specificities, enabling a visualization of the functional trait in subpopulations across the borders of species and genera. Such a quantitative insight into the niche separation of flavobacterial taxa complements our understanding of the ecology of polysaccharide-degrading bacteria beyond omics-based techniques on a single-cell level.

## Introduction

Glucuronomannan is a universal polysaccharide found in diatom cell walls. Its backbone consists of α-1,3-mannose alternating with β-glucuronic acid residues and is often branched and sulphated [1–4]. Since diatoms are a major polysaccharide source and contribute substantially to the marine food web by attributing to 40% of primary production [5–7], it can be assumed that glucuronomannan is widely distributed wherever diatoms are found. Hence, marine bacteria are adapted to its degradation and, especially *Bacteroidetes*, can digest substrates rich in sulphated α-mannose using complementary carbohydrate active enzymes (CAZymes), such as glycoside hydrolases (GHs) of family 92 [8]. The GH92 family codes for exo-acting α-mannosidases and thus far, catalytic activity has been detected on α-1,2-, α-1,3-, α-1,4- and α-1,6-linked mannose [9]. GH92 genes are common in many flavobacterial species. Therefore, a co-evolution along with diverse cell wall components of algae seems plausible and would explain the many GH92 variants. Extensive analysis of metagenome and metaproteome data retrieved from bacterioplankton seawater samples revealed conserved GH92-rich polysaccharide utilization loci (PULs) that contain sulfatases and cluster for their SusC-like TonB-dependent transporters and SusD-like substrate-binding proteins (SusCD-like tandem pairs) [10]. Their specific combination of CAZymes strongly suggests a degradation potential for sulphated α-glucomannans such as glucuronomannan [11]. Hence, GH92s from these particular PULs may act in a concerted manner on the different linkage types of the glucuronomannan, maximizing cell wall degradation of diatoms. The highly similar sequences of these PULs were retrieved from species of the *Formosa* and *Polaribacter* genera, which recurrently dominated the bacteroidetal response to algae blooms in 2010–2012 in the North Sea [10]. It was also shown from bacterioplankton-derived (meta-) proteomic data, that the expression levels of GH92s were consistently high during phytoplankton spring blooms in the North Sea [12]. As well as being recurrent in bacterioplankton-derived metagenomic data from the North Sea [13], GH92 genes were also identified in samples from the North Atlantic Ocean [14], which underlines their ecological importance.

In this study, we investigated the in situ distribution of these conserved putative glucuronomannan PULs among various taxa in complex planktonic samples from a time series taken in the North Sea to visualize the hypothesis raised by the metagenomic analyses. To this end, we targeted the two most common variants of GH92 genes, GH92_a and GH92_b, in two representative strains of recurrently blooming flavobacterial clades isolated from the North Sea: *Formosa* Hel1_33_131 [15, 16] and *Polaribacter* Hel1_33_49 [15, 17, 18]. We applied direct-geneFISH [19], a molecular tool that uses fluorescence in situ hybridization (FISH), to directly link a taxonomic identity to a putative glucuronomannan degradation potential. The method identifies cells via 16S ribosomal RNA (rRNA)-targeted oligonucleotides and simultaneously detects the gene(s) of interest with double-stranded DNA polynucleotide probes (typical length of 300–500 bp), allowing the visualization of key genes directly within the bacterial host. Furthermore, we evaluated whether the specificity of gene-targeting polynucleotide probes can be broadened by relaxing the hybridization conditions. In contrast to oligonucleotide probes, which need to be applied under stringent conditions during hybridization to gain specific signals, polynucleotide probes tolerate a certain amount of mismatches without compromising specificity. This property can be used to detect genes within one species but also highly similar genes in closely related taxa. Consequently, applying direct-geneFISH at relaxed conditions would allow to analyze an environmental sample based on a functional trait rather than on taxonomic affiliation alone.

In combination with high-resolution microscopy, direct-geneFISH has the additional advantage of providing direct, visual evidence of individual lifestyles and a quantitative insight into niche separation, which extends beyond what can be predicted from metagenome studies. Investigating flavobacterial genetic repertoires and their potential to degrade polysaccharides in combination with their visualization can help to define the niche spaces of these recurring clades and to understand their contribution to the marine carbon cycle.

## Material and methods

### Plankton samples and representative pure cultures

Plankton samples were collected in the German Bight at Helgoland Roads, station ‘Kabeltonne’ (54° 11.3′ N, 07° 54.0′ E) in the framework of a time series as described in [12, 13]. We used subsamples from the fixed filters taken on 3rd April 2009, 20th and 26th April 2010, 26th April and 3rd May 2012. Pure cultures of *Formosa* Hel1_33_131 [15, 16] (UBA3537 according to Genome Taxonomy Database (GTDB) release 83 [20]) and *Polaribacter* Hel1_33_49 were previously obtained by dilution cultivation from the same sampling site on 20th April 2010 [15, 17]. Cells were grown in HaHa_100 medium [15], fixed in exponential phase with formaldehyde (2%) and filtered onto polycarbonate filters (0.2 μm pore size).

### Synteny of putative glucuronomannan PULs

**Fig. 1:**
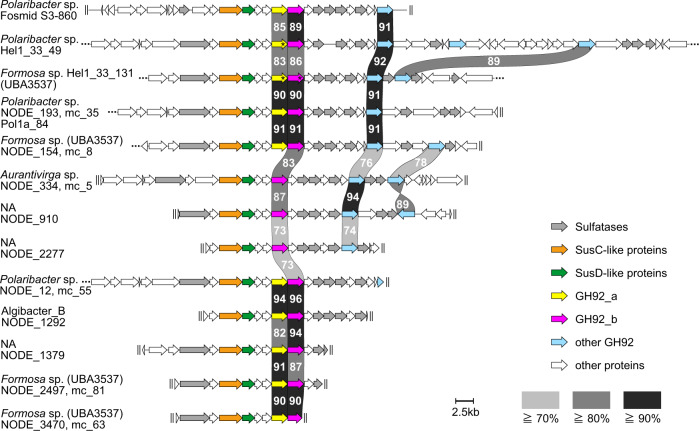
Synteny map of PULs likely encoding glucuronomannan degradation, retrieved from 2010–2012 Helgoland MAGs and isolates and a PUL found on a fosmid retrieved from the North Atlantic Ocean in 2006 [14]. The synteny map is aligned on the SusCD-like tandem pairs (SusC-like: orange, SusD-like: green). GH92_a are highlighted in yellow and GH92_b in magenta (coherent with the color-coding in following figures), all other GH92s in light blue. Sulfatases are displayed in gray. The synteny map was calculated with clinker [23] based on the amino acid sequences, but shown here are the corresponding nucleotide sequence identities. These numbers are only true for the immediate neighbors and are meant to showcase their remarkable similarity. PULs were sorted by complexity except for the PUL from the fosmid that was added for comparison at the top. Continuing contigs are indicated by “…” and end of contigs by “II”. The three GH92 genes for which polynucleotide probe sets were designed are marked with asterisks. NA = contig not assigned (binned) to a MAG, thus taxonomy unassigned.

For analysis of PUL synteny, we retrieved all putative glucuronomannan PULs from 38 metagenomes taken at station ‘Kabeltonne’ in the years 2010–2012 during spring phytoplankton blooms, published in [10]. They were annotated as described therein and defined by the existence of at least one *susC*- or *susD*-like gene and at least two degradative CAZymes from the glycoside hydrolase or polysaccharide lyase families and additionally by a combination of predicted GH92 and sulfatase genes. We included representatives even if they occurred only in a single metagenome. All remaining PUL contigs were annotated using Prokka v 1.12 [21] (with adaptions for the –c and –m options within Prodigal as described in [22]) with manual annotation of the predicted genes and shortened to the genetic region of the PULs. Their taxonomic affiliation was determined using the metagenome-assembled genome (MAG) affiliation from [10] and the respective taxonomic classification using the GTDB-Tk genome-based taxonomy (GTDB-Tk v0.0.8 with GTDB release 83 [20]). PULs from *Formosa* Hel1_33_131 and *Polaribacter* Hel1_33_49 genomes were extracted accordingly. Respective GenBank files for all shortened contigs and genomes are available as Supplementary Data 1. The synteny map of the PULs in Fig. 1 was calculated with clinker to directly visualize the structural similarity on the protein level using GenBank files as input [23].

### Phylogenetic tree of *Flavobacteriaceae* in Helgoland metagenomes

To visualize the distribution of glucuronomannan PULs within the family of *Flavobacteriaceae*, we constructed a reduced tree (Supplementary Fig. 1) based on the phylogenetic tree published in [10] using only sequences classified as *Flavobacteriaceae*. The tree was computed as in [10] with the exception of using RAxML v8.2.11 [24]. It was visualized using iTOL [25].

### GH92 identities and taxonomic affiliation of non-PUL GH92s

Nucleotide and amino acid identity values for all GH92 genes on representative glucuronomannan PULs were calculated using multiple sequence alignments computed with MAFFT v7.017 [26] with E-INS-I algorithm. Pairwise identities and stretches of mismatches or gaps of each polynucleotide probe (FORM-GH92_a_1-5, FORM-GH92_b_1-5 and POL-GH92_a_1-5) to the corresponding GH92_a or GH92_b in PULs of Fig. 1 were calculated using Nucleotide-Nucleotide BLAST [27] within Geneious 8.1.8 (https://www.geneious.com) (Supplementary Table 1). To check for the presence of GH92 genes in the 38 bacterioplankton metagenomes that were not located within one of the putative glucuronomannan PULs but maintained a nucleotide sequence similarity of 80% or higher, we compared all sequences of the designed gene-targeted polynucleotide probes to all predicted GH92s in the metagenomes using Nucleotide-Nucleotide BLAST v2.8.1 [27]. A hit was accepted if at least four of five nucleotide probes from a set had a hit with ≧80%. Taxonomic affiliation of these hits was again determined using the MAG affiliation from [10] and the respective taxonomic classification using the GTDB-Tk genome-based taxonomy (Supplementary Table 2).

### Oligonucleotide 16S rRNA probe design

All 16S rRNA oligonucleotide probes are summarized in Supplementary Table 3 and were ordered directly labeled with four fluorescent dyes from biomers.net GmbH (Ulm, Germany) that are specified in Supplementary Table 4. In this study, new helper oligonucleotides that flank the 16S rRNA probe FORM181B on either side were designed with ARB software [28] on the Silva SSURef NR 99 v128 16S rRNA sequence database [29], to improve accessibility for the probe to its target sequence.

### Polynucleotide probes

For the design of the GH92-targeted polynucleotide probe sets, the published genomes of *Formosa* Hel1_33_131 (accession number in NCBI’s Genbank: CP017260.1) and *Polaribacter* Hel1_33_49 (JPDI00000000) were used. The three most widely distributed GH92 genes in our metagenome analyses served as targets for direct-geneFISH: FORM-GH92_a (gene locus tag: FORMB_02890, protein_id: AOR27350.1), FORM-GH92_b (gene locus tag: FORMB_02900, protein_id: AOR27351.1) and POL-GH92_a (gene locus tag: PHEL49_1329, protein_id: KGL60439.1). For each gene, a set of five consecutive polynucleotide probes of 442-456 bp length and their respective primer sets were designed with SnapGene® software (from GSL Biotech; available at snapgene.com) (Supplementary Table 5, Supplementary Data 2). The polynucleotide probes within each set were designed to have a similar melting temperature (Tm) and GC content to enable simultaneous hybridization. To investigate the stringency boundaries that determine the probe specificities in direct-geneFISH, we used the Wetmur formula for DNA:DNA hybrids, where [Na^+^] = *molar Na*^+^ concentration, GC = GC content [%], N = probe length [bp], M = mismatch [%] and FA = formamide concentration [%], [30]:

$${{{{{{{\mathrm{Tm}}}}}}}} = 81.5 + 16.6\,{{{{{{{\mathrm{log}}}}}}}}10\left( {\frac{{\left[ {{{{{{{{\mathrm{Na}}}}}}}}^ + } \right]}}{{1 + 0.7\left[ {{{{{{{{\mathrm{Na}}}}}}}}^ + } \right]}}} \right) + 0.41\,{{{{{{{\mathrm{GC}}}}}}}} - \left( {\frac{{500}}{{{{{{{{\mathrm{N}}}}}}}}}} \right) - {{{{{{{\mathrm{M}}}}}}}} - 0.63\,{{{{{{{\mathrm{FA}}}}}}}}$$

For each polynucleotide probe, the corresponding melting temperatures were calculated across varying parameters, such as formamide (FA) concentration and the respective allowed percentage of mismatch for the probe to the target DNA.

The polynucleotide probes were synthesized as described in [19] and either individually labeled or pooled in equi-molar probe mixes and then labeled with the Thermo Fisher Ulysis™ Alexa Fluor™ Nucleic Acid Labeling Kit (catalog numbers: Alexa Fluor^®^ 488: U21650, Alexa Fluor^®^ 594: U21654, Alexa Fluor^®^ 647: U21660), according to the manufacturer’s instructions with the following modifications: The amount of dye added to 1 μg DNA was increased to 3 μl for Alexa Fluor^®^ 488, 15 μl for Alexa Fluor^®^ 594 and 10 μl for Alexa Fluor^®^ 647, which successfully enhanced the degree of labeling (number of dyes per 100 bp) (Supplementary Table 6). The time for the labeling reagent to react with the probe DNA was increased to 30 min. Unbound dyes were removed with Bio-Spin™ P-30 Gel Columns (Tris Buffer, catalog number: 7326223, Bio-Rad, Hercules, California, USA).

As negative controls, we used polynucleotide probes that target a gene of unknown function (*unk*) originating from a *Pseudoalteromonas* phage PSA-HP1 (synthesized as described in [31], displayed as negative control (NC) in the figures), as well as hybridizations without any added gene probes (data not shown). To confirm that the *unk*-targeted polynucleotide probes used as NC did not hybridize to any DNA in the plankton samples, their probe sequences were blasted against all predicted genes within the analyzed 38 metagenomes and no hits were detected.

### Direct-geneFISH

Direct-geneFISH was performed as described in [19] with a few modifications. The permeabilization step was omitted for *Formosa* Hel1_33_131 to preserve cell morphology, even though it decreased the detection efficiency for the gene slightly. Permeabilization of *Polaribacter* cells was performed for 5–10 min at room temperature with 0.5 mg ml^*−*1^ lysozyme in buffer (1× PBS, 0.05 M ethylenediaminetetraacetic acid (EDTA), 0.1 M Tris-HCl). Hybridized samples were counterstained with 4′,6-diamidino-2-phenylindole (DAPI) and mounted with ProLong™ Gold Antifade Mountant (catalog number: P36930, Thermo Fisher Scientific) on a microscope slide, covered with a #1.5 coverslip and cured for 24 h at room temperature.

### Microscopy

The Airyscan detector upgrade on a confocal laser scanning microscope Zeiss LSM 780 was run in super-resolution mode (63× plan apochromatic oil immersion objective, 32 GaAsP detectors) [32]. The excitation lasers and emission detection windows are listed in Supplementary Table 7. For each field of view, a z-stack was taken and after post-processing with default Airyscan filtering, a 3D reconstruction was performed with Zen Black 2.1 software (Version 13.0.0.0, Carl Zeiss, Germany) with automatic Airyscan filter strength. The final images were obtained by a maximum intensity projection of the z-stack. The histogram of signal intensities was adjusted to maximize visibility of gene probe-conferred positive signals and the same values were used as thresholds to evaluate negative controls of the respective experiment to estimate eventual false positives. For better representation, fluorescent signals were consistently pseudocoloured, not resembling their original labeling dye color.

### Gene detection efficiencies and relative gene abundances

For each direct-geneFISH polynucleotide probe set, we determined the gene detection efficiency under stringent hybridization conditions on pure cultures. Per experiment, we analyzed 3–8 fields of view in which 721–2747 cells were counted. The efficiencies are given as the mean of the percentages of gene-positive cells ((number of cells with gene signal/total number of cells) * 100) of each evaluated field of view and the corresponding standard deviation. In plankton samples, we report the fraction of 16S rRNA-targeted cells carrying a gene signal as the relative gene abundances (RGAs) in [%] and the corresponding standard deviation. Only cells with both a 16S rRNA-FISH and DAPI-DNA signal were considered for counting. Per experiment on plankton samples, we analyzed 6–20 fields of view in which 41–387 cells were counted. More details, such as the total number of cells counted per experiment, are given in Supplementary Table 4. *P*-values for evaluating differences in efficiencies were calculated using a two-tailed *t*-test assuming equal variances (confirmed by two-sample *F*-test for variances) in Excel.

## Results

### Synteny of glucuronomannan PULs

We adapted direct-geneFISH to target genes from conserved, putative glucuronomannan-degrading PULs in the flavobacterial groups *Polaribacter* and *Formosa*. A preceding analysis of (meta-) genomic data obtained from sampling campaigns in the German Bight in 2010–2012 indicated that the gene sequences of *susCD*-like tandem pairs retrieved from GH92- and sulfatase-rich PULs, clustered together based on their amino acid sequence similarity [10]. A more detailed analysis revealed that these PULs were not only taxonomically linked to representative strains and MAGs of the genera *Formosa* and *Polaribacter* [10], but also to MAGs affiliated to *Aurantivirga* and *Algibacter* (Fig. 1, Supplementary Fig. 1). We examined the synteny of these PULs and found that they share a high sequence identity for their SusCD-like proteins, GH92s and sulfatases, within and across genera (Fig. 1). Furthermore, we could confirm that the PULs share similarly high sequence identity with a PUL found on a fosmid affiliated to *Polaribacter* sp. (fosmid S3-860) retrieved from bacterioplankton samples taken in the North Atlantic Ocean in 2006 [14]. GH92 genes at the same positions within the PULs are homologous across strains, sharing a high percentage of sequence identity (Fig. 1). For example, FORM-GH92_b and POL-GH92_b share a nucleotide sequence identity of 86% (Fig. 1, Supplementary Table 8). However, the sequence comparison of the different GH92 genes within the same PUL showed they have less than 50% nucleotide sequence identity, most likely reflecting differences in substrate specificity of these exo-α-mannosidases. When compared to GH92 protein sequences within the CAZy database [33], no discrete specificity could be determined for GH92_a, while the closest characterized proteins to GH92_b and GH92_c were α-1,3-mannosidases. GH92_d and GH92_e clustered with α-1,2-mannosidases. Interestingly, while a homolog of GH92_b is present in all and of GH92_a in most of the putative glucuronomannan PULs in Fig. 1, other GH92 variants were found only sporadically, which could also be due to incomplete assemblies of the respective MAGs.

### Gene detection efficiency of each gene-targeting probe set

**Fig. 2:**
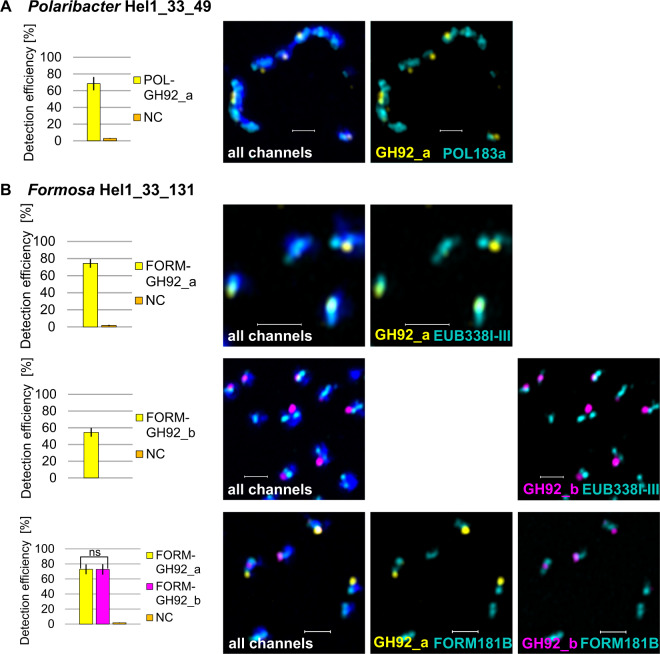
Detection efficiencies of three probe sets targeting GH92 genes with direct-geneFISH in pure cultures of *Polaribacter* Hel1_33_49 and *Formosa* Hel1_33_131. **A** POL-GH92_a in *Polaribacter* was targeted with Alexa 488-labeled polynucleotide probes (yellow) and the 16S rRNA with the species-specific probe POL183a (cyan). **B** FORM-GH92_a in *Formosa* was targeted with Alexa 594-labeled polynucleotide probes (yellow), FORM-GH92_b with Alexa 647-labeled polynucleotide probes (magenta) and the 16S rRNA with a species-specific probe FORM181B or general bacterial probe EUB338I-III (cyan). The DNA was counterstained with DAPI (appears blue in “all channels”). Shown micrographs are maximum intensity projections of processed images achieved with Airyscan microscopy in super-resolution mode. NC = negative control, ns = not significant, scale bar: 1 µm, error bars in histograms represent standard deviations of the mean efficiencies. Experimental details are given in Table 1 and Supplementary Table 4.

For each gene-targeting polynucleotide probe set, we determined the gene detection efficiencies under stringent conditions in pure cultures of *Polaribacter* and *Formosa*. We aimed to target the most widely distributed GH92s in our metagenome analyses. The polynucleotide probe sets were designed based on the genomes of two representative strains to target the genes POL-GH92_a in *Polaribacter* Hel1_33_49 and FORM-GH92_a and FORM-GH92_b in *Formosa* Hel1_33_131. Each set consists of five adjacent probes between 442–456 bp in length (Supplementary Table 5). When blasted against each other, the longest stretch of mismatches between single probes and the target DNA from respective GH92 genes within examined PULs was 12 bp (Supplementary Table 1). The pairwise identity of each probe compared to the equivalent GH92 variant in Fig. 1 ranged between 73 and 100%, with an average of 88% (Supplementary Table 1). Per probe set, we could incorporate between 38 and 77 dyes (details in Supplementary Table 6). We performed direct-geneFISH successfully on pure cultures of *Polaribacter* Hel1_33_49 and for the probe set POL-GH92_a, we achieved a detection efficiency of 68.4%  ± 7.9% (negative control (NC): 2.9% ± 0.4%) (Table 1, Fig. 2A). Additionally, we targeted the genes FORM-GH92_a and FORM-GH92_b individually in *Formosa* Hel1_33_131, which resulted in similar efficiencies: 74.3% ± 5.0% (NC: 1.8% ± 0.7%) and 54.5% ± 5.2% (NC: 0.0% ± 0.0%), respectively. Detecting both genes simultaneously in a double gene hybridization, the efficiencies reached 72.8% ± 6.5% for FORM-GH92_a and 72.7% ± 6.9% for FORM-GH92_b (NC: 1.8% ± 0.4%) (Table 1, Fig. 2B). For targeting the 16S rRNA, we used either species-specific probes (POL183a or FORM181B) or the general bacterial probe EUB338I-III.

**Table 1:** Summary of the highest mean efficiencies and their corresponding standard deviations for the GH92 gene detection in pure cultures, plankton samples and direct-geneFISH experiments for broadening the probe specificities across genera.

Relative 16S rRNA abundances are based on CARD-FISH counts [13, 18]. All GH92_a signals are pseudocoloured in yellow in the following figures, all GH92_b signals in magenta and all 16S rRNA signals in cyan. # FOVs = number of evaluated fields of view. HC = hybridization conditions, S = stringent, R = relaxed. More experiments and other details are listed in Supplementary Table 4.

### Detection of relative gene abundance in plankton samples

**Fig. 3:**
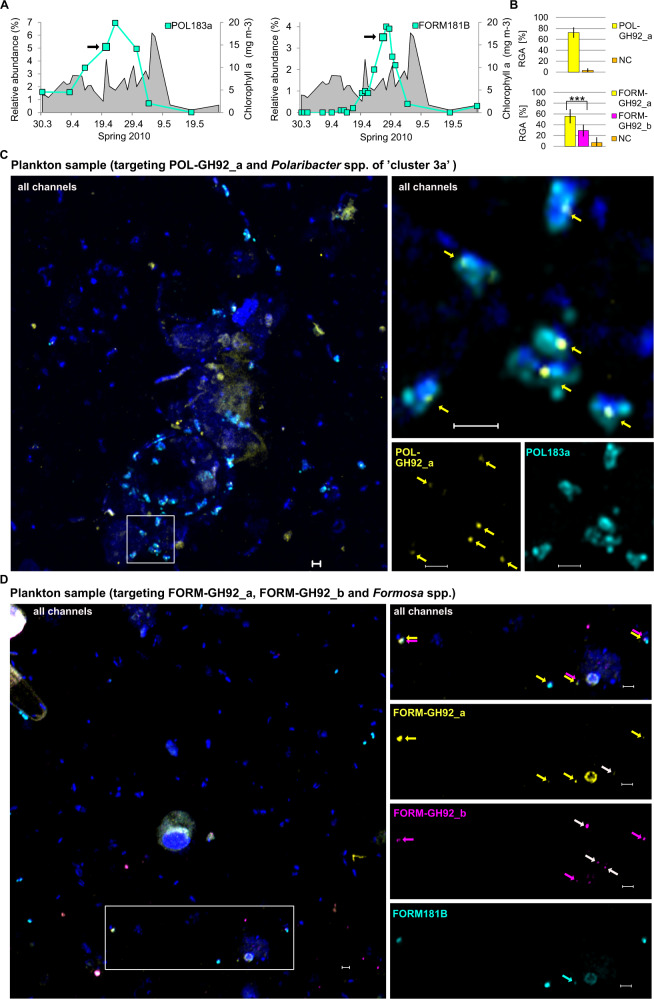
Detection of GH92 genes with direct-geneFISH in plankton samples taken during the spring bloom in the German Bight. **A** Relative cell abundances of *Polaribacter* spp. of “cluster 3a” targeted with the 16S rRNA probe POL183a and *Formosa* spp. targeted with the 16S rRNA probe FORM181B based on CARD-FISH counts [13]. Chlorophyll a concentration was taken as a proxy for algae biomass (gray area). Black arrows indicate dates from which samples were used for experiments (left: 20th April 2010, right: 26th April 2010). **B** Relative gene abundance (RGA), NC = negative control, error bars in histograms represent standard deviations of mean RGAs. Significance is indicated by asterisks (*p* ≤ 0.001). **C** Overview image to the magnified regions of interest to the right of *Polaribacter* spp. of “cluster 3a” targeted with the 16S rRNA probe POL183a and POL-GH92_a in a plankton sample. **D** Overview image to the magnified regions of interest to the right of *Formosa* spp. targeted with the 16S rRNA probe FORM181B and FORM-GH92_a and _b in a plankton sample. DNA was counterstained with DAPI (appears blue in ‘all channels’). Arrows in magenta and yellow show the respective gene signals and white arrows indicate gene-like signals in non-target organisms. Arrow in cyan points to a cell with a low ribosome content. All micrographs are maximum intensity projections of processed images achieved with Airyscan microscopy in super-resolution mode. Scale bar: 1 μm.

In bacterioplankton samples collected during the phytoplankton spring bloom in the German Bight near Helgoland in 2010 relative abundances of GH92 genes were determined with direct-geneFISH. Based on catalyzed reporter deposition (CARD)-FISH counts, the relative abundance of cells affiliated to *Polaribacter* spp. “cluster 3a”, with the 16S rRNA-targeted probe POL183a, was 5.1% on 20th April 2010 (Fig. 3A, [18]). “Cluster 3a” was only detected in metagenomic reads from 2010 and includes the strain *Polaribacter* Hel1_33_49. The RGA of gene POL-GH92_a detected in DAPI-stained cells with a positive 16S rRNA probe signal (POL183a), was comparable to the gene detection efficiency in cultured representatives: 72.5% ± 9.6% (NC: 3.0% ± 3.8%) (Table 1, Fig. 3B). The relative abundance of *Formosa* cells (FORM181B) was 3.5% on 26th April 2010 (Fig. 3A) [13]. The RGAs for genes FORM-GH92_a and FORM-GH92_b were 55.7% ± 13.0% for FORM-GH92_a and 29.2% ± 11% for FORM-GH92_b (NC: 6.9% ± 2.1%) (Table 1, Fig. 3B). In plankton samples taken on 3rd April 2009, we obtained RGAs of 42.1% ± 29.5% for FORM-GH92_a and 31.9% ± 24.8% for FORM-GH92_b (NC: 4.2% ± 12.5%). More detailed information and additional experiments can be found in the Supplementary Text and Supplementary Table 4.

### Broadening polynucleotide probe specificity to include close relatives

**Fig. 4:**
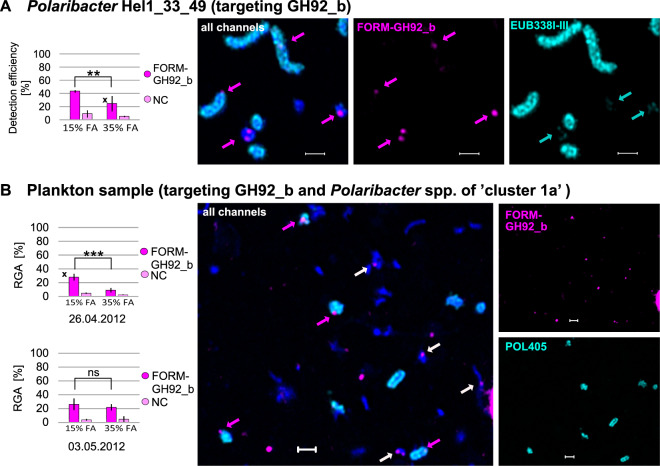
**A** Detection of GH92_b with FORM-GH92_b gene probe set in pure culture of *Polaribacter* Hel1_33_49 targeted with general bacterial 16 S rRNA probe EUB338I-III. **B** Detection of GH92_b with FORM-GH92_b gene probe set in *Polaribacter* spp. of “cluster 1a” (targeted with 16S rRNA probe POL405) in plankton samples taken in the German Bight on the 26th April 2012 and 3rd May 2012. Significance levels are indicated by asterisks (***: *p* ≤ 0.001, **: *p* ≤ 0.01), ns = not significant. Arrows in magenta show the detected gene signals, white arrows display gene-like signals in other non-target organisms, arrows in cyan point to cells that have a low ribosomal content. Micrographs display experiments marked with a cross in the bar plots. Shown micrographs are maximum intensity projections of processed images achieved with Airyscan microscopy in super-resolution mode. DNA was counterstained with DAPI (appears blue in ‘all channels’). RGA = relative gene abundance, NC = negative control, scale bar: 1 μm, error bars represent the standard deviations.

For an application on environmental samples, we aimed to broaden polynucleotide probe specificities to allow for the detection of similar genes beyond the borders of species and across genera. This was achieved by carefully relaxing the stringency during hybridization. The homologous genes FORM-GH92_b from *Formosa* and POL-GH92_b from *Polaribacter* share a nucleotide sequence identity of 86% (Supplementary Table 8). Theoretically, the gene POL-GH92_b can be detected with the probe set designed for FORM-GH92_b by relaxing the hybridization conditions and adjusting the FA concentration within the hybridization buffer from 35% to 15% (Supplementary Table 9). To test this hypothesis, we performed direct-geneFISH with the FORM-GH92_b gene probe set on pure cultures of *Polaribacter* Hel1_33_49 with hybridization buffers containing 35% and 15% FA. The GH92_b genes in *Polaribacter* were detected with an efficiency of 43.6% ± 1.9% (NC: 9.3% ± 5.5%) at relaxed conditions (15% FA) and with a significantly lower efficiency of 24.8% ± 11.6% (NC: 5.4% ± 1.0%) at stringent conditions (35% FA) (*p* < 0.01, Fig. 4A, Table 1). These results confirmed that the target specificity of the polynucleotide probe sets can be broadened by relaxing the stringency of hybridization conditions.

From the study of Avcı et al. [18], we know that *Polaribacter* spp. of “cluster 3a” were only abundant in the year 2010, whereas *Polaribacter* spp. of “cluster 1a” were abundant in 2012. “Cluster 1a” consists of four MAGs that are targeted by the 16S rRNA probe POL405, but only POL1a_84 harbors a putatively glucuronomannan degrading PUL [18] that clusters with the other PULs shown in Fig. 1. The GH92_b gene of MAG POL1a_84 has a nucleotide percentage identity of 87.7% with POL-GH92_b from *Polaribacter* Hel1_33_49 and 90.3% with FORM-GH92_b from *Formosa* Hel1_33_181 (blast analysis in Supplementary Table 8). Therefore, we combined the FORM-GH92_b probe set and the 16S rRNA probe POL405 to target “cluster 1a” and the respective GH92_b gene in two samples taken during the spring phytoplankton bloom in the German Bight in 2012 (Table 1, Fig. 4B). Indeed, we were able to detect the GH92_b gene from MAG POL1a_84 on 26th April 2012 with a relative abundance of 28.0% ± 5.0% (NC: 4.6% ± 1.0%) at relaxed conditions and with 9.1% ± 3.2% (NC: 2.5% ± 0.6%) at stringent conditions (*p* < 0.001). On 3rd May 2012, we could detect the gene with an RGA of 26.2% ± 8.4% (NC: 3.8% ± 1.3%) at relaxed conditions and with 21.9% ± 4.6% (NC: 4.7% ± 4.2%) at stringent conditions (*p* > 0.05). This is particularly interesting, since the relative abundance based on CARD-FISH counts was much lower on the second date (1.8%) than on the first date (12.7%) and yet, successful gene detection was possible.

### In situ localization

By combining direct-geneFISH with high-resolution microscopy, we determined whether cells carrying a GH92 gene are rather particle-associated or free-living. On plankton samples from the German Bight of two consecutive years, only 18.6% ± 18.6% (3rd April 2009) and 19.4% ± 10.2% (26th April 2010) of cells identified with 16S rRNA probe FORM181B were attached to algal debris (Fig. 3D). In contrast, 58% ± 21.9% of cells of *Polaribacter* “cluster 3a” were attached to algal-like remnants on samples from 20th April 2010 (Fig. 3C, Supplementary Figs. 2, 3). Cells of *Polaribacter* “cluster 1a” however, were rather found free-living. In samples from two dates in 2012, only 13% ± 5.7% (26th April 2012) and 29% ± 7.1% (3rd May 2012) of the cells were attached to algae debris (Fig. 4B, Supplementary Fig. 4). The microscopic analysis also provided the opportunity to determine whether GH92 genes were over- or underrepresented in either the attached or the free-living fraction of cells, but both genes were nearly evenly distributed among both fractions (data not shown).

## Discussion

In this study, we targeted the two most common GH92 variants from conserved and putatively glucuronomannan degrading PULs with direct-geneFISH. With an efficiency of up to 74%, we report, so far, the highest numbers for the detection of single-copy genes in pure cultures and environmental samples with (direct-) geneFISH (compare for example [19, 34, 35]).

After successful tests on two flavobacterial pure cultures, *Polaribacter* Hel1_33_49 and *Formosa* Hel1_33_131, we examined plankton samples from the German Bight taken in 2010. We could show that almost three quarter of *Polaribacter* individuals of “cluster 3a” carried the POL-GH92_b gene, reaching the same numbers as in pure culture experiments. More than half of all cells in plankton samples identified as *Formosa* sp. carried the FORM-GH92_a gene but only one third the FORM-GH92_b gene. These differences, however, were not a result of strain variation within *Formosa* spp., evidenced by the even distribution of read mapping coverage for both genes and the switching of the dyes for the gene-targeting probes that resulted in RGAs not differing significantly in gene proportions (see Supplementary Text for details). Therefore, we do not think that the different gene hybridization pattern represents a biological pattern, but suggest it is rather based on the delicate cells of *Formosa*. During direct-geneFISH, *Formosa* cells tend to break and leak cell content, which could lead to some loss of geneFISH signals (some cell disruption is also visible in Fig. 2B).

Even though it extends beyond the scope of this study, we hypothesize that the high similarity of our conserved glucuronomannan PULs that are unevenly distributed among four genera (Supplementary Fig. 1) suggests a horizontal gene transfer between them. Other studies already showed that CAZymes and entire syntenic PULs are commonly transferred between both closely and distantly related bacteroidetal species [36–39]. To detect possible gene variants across the borders of species and genera with direct-geneFISH, it is necessary to relax the hybridization conditions due to the less conserved nature of the targeted genes compared to the ribosomal RNA (see also [40]). Indeed, we could show that by relaxing the stringency of hybridization, the probe sets can be used to detect genes with up to 20% mismatches. Just recently, it was similarly shown in pure cultures that the detection of genes with mismatches of up to 20% is possible [34] using the original CARD-geneFISH protocol [35] and a single digoxigenin-labeled polynucleotide probe with antibody amplification, proving the robustness of this approach. Taking this to the next step, we successfully tested our hypothesis on North Sea plankton samples taken in 2012: Almost 30% of cells affiliated to *Polaribacter* sp. from “cluster 1a” carried the GH92_b gene detected with the *Formosa*-specific probe set FORM-GH92_b (Fig. 4B). Our experiments prove that by modulating the stringency via the FA-concentration, direct-geneFISH can provide an in situ link of a functional trait even to a subpopulation of yet uncultured *Polaribacter* spp. in plankton samples of which we only have evidence from metagenomic data. Our findings open up new perspectives for the detection and visualization of functional guilds that share a similar trait and degradation potential in their genomes but belong to different taxa [41]. To narrow down the identity of the host carrying the functional genes, the use of multiple polynucleotide probes with high signal intensities in combination with mild cell-fixation can be sufficient for fluorescence-activated flow cytometric cell sorting based on target genes. Sorted cells carrying the functional gene of interest can then be subjected to genome amplification and sequencing for taxonomic identification [42].

Micrographs of our plankton samples also displayed cells that showed gene-like signals without a corresponding 16S rRNA signal. Such signals could derive from bacterioplankton species from the four genera of *Formosa*, *Polaribacter*, *Aurantivirga,* and *Algibacter* that harbor that particular gene (or a highly similar version of it) but are not identified by the applied 16S rRNA probe (Supplementary Fig. 1). From the relative abundances of *Formosa* and *Polaribacter* spp. over the course of the spring bloom in 2010, we know that they were present on both sampling dates (Fig. 3A) and that their GH92s can be detected across genera with a smaller but yet pronounced detection rate (Fig. 4A). Therefore, the white arrows in Fig. 3D might point to cells which are affiliated to *Polaribacter* spp. that also carry the target gene. Another example can be seen in Fig. 4B, where GH92_b genes were detected in samples from 26th April 2012. The relative cell abundances were 12.7% for the 16S rRNA targeted probe POL405 (“cluster 1a”), 0.5% for POL183a (“cluster 3a”) and 0% for FORM181B [13, 18]. Thus, the white arrows in Fig. 4B most likely point to cells of “cluster 3a” that were also present on that date. However, blast analysis of all polynucleotide gene probe sequences against predicted GH92 genes in the spring bloom metagenomes yielded additional GH92 variants which are not encoded for in conserved glucuronomannan PULs, like those defined for Fig. 1. Still, they share a sequence similarity ≧80% and could be targeted by one of our probes sets (Supplementary Fig. 1, Supplementary Table 2). The GH92-containing contigs that were identified, taxonomically belonged exclusively to the four genera of *Formosa*, *Polaribacter*, *Aurantivirga*, and *Algibacter* and most of them even to the corresponding species or clusters shown in Fig. 1.

Combining direct-geneFISH with high-resolution microscopy also provides indications on the lifestyles of the targeted bacterioplankton clades. Earlier studies suggested a free-living lifestyle for *Formosa* clade A and B (Hel1_33_131), due to their rather small genome size [15], in contrast to the related *Formosa agariphila* for which an attached lifestyle was suggested [43]. Indeed, after thorough microscopic analysis of plankton samples taken from the German Bight in 2010, we confirmed that only 20% of the individual cells identified with 16S rRNA probe FORM181B were attached to algal debris (Fig. 3D). The evaluation of micrographs from the same spring bloom showed a strikingly different scheme for cells from *Polaribacter* “cluster 3a” (POL183a): 60% were, on average, attached to algal-like remnants (Fig. 3C, Supplementary Figs. 2,  3), even though previous studies suggested a free-living lifestyle due to their genome size and limited enzyme repertoire [17]. In contrast to that, the MAGs of *Polaribacter* “cluster 1a” harbor an extensive repertoire of CAZymes and PULs associated with the utilization of nine different polysaccharides, suggesting a specialization for growth on microalgae and brown algae with a potential attached lifestyle [18]. “Cluster 1a” includes several species-representative MAGs which is reflected in the different morphologies of the FISH-stained cells, ranging from coccoid to rod shaped (Supplementary Fig. 4). From the micrographs, we could further infer that, while being high in relative abundance on 26th April 2012 with 12.7%, only 13% of “cluster 1a” cells were attached to algae debris (Fig. 4). This was in stark contrast to their distribution only days later, when the relative abundance dropped to 1.8% on 3rd May 2012 and proportions of cells attached to algae and their remnants more than doubled. Our microscopy analysis of plankton samples suggests that even though the examined *Formosa* and *Polaribacter* spp. share the glucuronomannan-degradation potential encoded in conserved PULs, they appear to occupy different niche spaces in the same habitat.

In future experiments, it would be promising to also microscopically co-localize the polysaccharide, which is putatively degraded, by staining with specific fluorescently-labeled antibodies or carbohydrate binding modules [44] in combination with direct-geneFISH to visualize the CAZyme-encoding gene. We can envision a reliable application on sediment or soil samples if critical steps, such as cell permeabilization, are adapted beforehand. Also, the background autofluorescence of such samples has to be considered and either bleached or tackled with a careful choice of fluorophores and corresponding embedding medium. For example, Rahlff et al. [45] already performed direct-geneFISH on biofilms without any major alterations of the protocol.

We have shown that we can reliably detect GH92 genes with direct-geneFISH in pure cultures and plankton samples, visually linking function and phylogeny even across the borders of genera. Our results emphasize the importance to not only analyze metagenomic data but also to integrate FISH-based methods and (high-resolution) microscopy for visual evidence of individual lifestyles of defined bacterial clades.

## Supplementary information

Supplementary Material

Supplementary Table 4

Supplementary Data 1

Supplementary Data 2
