# Supplementary material for: In situ visualization of glycoside hydrolase family 92 genes in marine flavobacteria
Source: ISME Commun. 2021 Dec 18;1:81. doi: 10.1038/s43705-021-00082-4 (PMC9723552; doi:10.1038/s43705-021-00082-4)
Supplement: Supplementary file 1 — Supplementary Material [file 43705_2021_82_MOESM1_ESM.pdf]

Supplementary Material

Supplementary Text

Detection of FORM-GH92_a and FORM-GH92_b in pure cultures and plankton samples

On 26th April 2010, the relative abundances of FORM-GH92_a and FORM-GH92_b genes in cells with a 16S rRNA probe signal (FORM181B) and a DAPI staining were 55.7% ± 13.0% and 29.2% ± 11% (NC: 6.9% ± 2.1%) (Table 1, Figure 3B). In contrast to the even proportions of both gene in pure cultures, the relative abundance for gene FORM-GH92_a was significantly higher than for gene FORM-GH92_b ($p \leq 0.001$). To test whether this uneven occurrence represents a biological pattern, we mapped the reads of the two metagenomes (taken three days before 26th April 2010 and four days afterwards, displaying similarly high CARD-FISH abundances of 2% and 2.2% (Teeling et al., 2016)) to the respective GH92 genes (data not shown). The very similar read mapping coverage values for FORM-GH92_a and FORM-GH92_b indicated that both genes were evenly distributed. Additionally, we synthesized new probe sets and switched the dyes that were used for labelling to test whether the uneven gene abundances were an artefact introduced by the dyes (for example their photostability). Two experiments on pure cultures of *Formosa* Hel1_33_131 were performed with these new probe sets, resulting in detection efficiencies of first, 61.8% ± 5.4% for FORM-GH92_a and 60.0% ± 8.0% for FORM-GH92_b (NC: 2.8% ± 2.0%) and secondly, 54.7% ± 3.1% for FORM-GH92_a and 49.5% ± 9.1% for FORM-GH92_b (NC: 1.8% ± 0.4%). For both experiments the differences of the efficiencies detection FORM-GH92_a and FORM-GH92_b are not significant. The efficiencies are evenly proportioned and in the same range as in the previous experiments (compare Supplementary Table 4). In plankton samples taken on 3rd April 2009, we obtained RGAs of 42.1% ± 29.5% for FORM-GH92_a and 31.9% ± 24.8% for FORM-GH92_b (NC: 4.2% ± 12.5%). These abundances are in the same range and not significantly different in proportion ($p \geq 0.05$) to previously obtained RGAs.

Supplementary Data

Supplementary Data 1 [GH92_PULs.gbk]
GenBank files for all shortened contigs and genomes that were used for the calculation of the PUL synteny map in Figure 1.

Supplementary Data 2 [polynucleotideprobe_sequences.fasta]
DNA sequences of all 15 gene-targeted polynucleotide probes.

Supplementary Figures and Tables

Supplementary Figure 1
Radial phylogenetic tree of *Flavobacteriaceae* in which the Mash cluster are highlighted that harbours the targeted GH92s either in a PUL displayed in Figure 1 or with at least a nucleotide identity of $\geqq 90$ or of $\geqq 80\%$ and $< 90\%$

Supplementary Figure 2
Detection of GH92_a genes in *Polaribacter* spp. of 'cluster 3a' (POL183a) with direct-geneFISH in plankton samples taken during the spring bloom in the German Bight on 26th April 2010.

Supplementary Figure 3
Detection of GH92_a in *Polaribacter* spp. of 'cluster 3a' (POL183a) with direct-geneFISH in a plankton sample taken on 20.04.2010 in the German Bight. Micrographs showing plankton sample with both, computationally suppressed autofluorescence and with autofluorescence.

Supplementary Figure 4
Exemplary micrographs of GH92_b detection with FORM-GH92_b gene probe set in *Polaribacter* spp. of 'cluster 1a' (POL405) with direct-geneFISH in a plankton sample taken on 3rd May 2012, displaying different cell morphologies.

Supplementary Table 1
Pairwise identity and longest stretch of mismatches between each of the 15 polynucleotide probes and the equivalent GH92-variants from the analysed putative glucuronomannan PULs in Figure 1.

Supplementary Table 2
Taxonomical classification of gene hits that are targeted by our gene probes but are not found in PULs as specified in Figure 1.

Supplementary Table 3

Oligonucleotide probes used in this study to target the 16S rRNA for cell identification.

Supplementary Table 4

Experimental details, detection efficiencies and RGAs for all executed experiments.

Supplementary Table 5

Polynucleotide probes and respective primer design.

Supplementary Table 6

Labelling details for polynucleotide probes used in this study.

Supplementary Table 7

Settings for Airyscan microscopy.

Supplementary Table 8

Nucleotide sequence identities of all GH92 genes in putative glucuronomannan PULs from Figure 1.

Supplementary Table 9

Mismatches calculated for relaxed and stringent hybridization conditions of polynucleotide probes FORM-GH92_b_1-5.

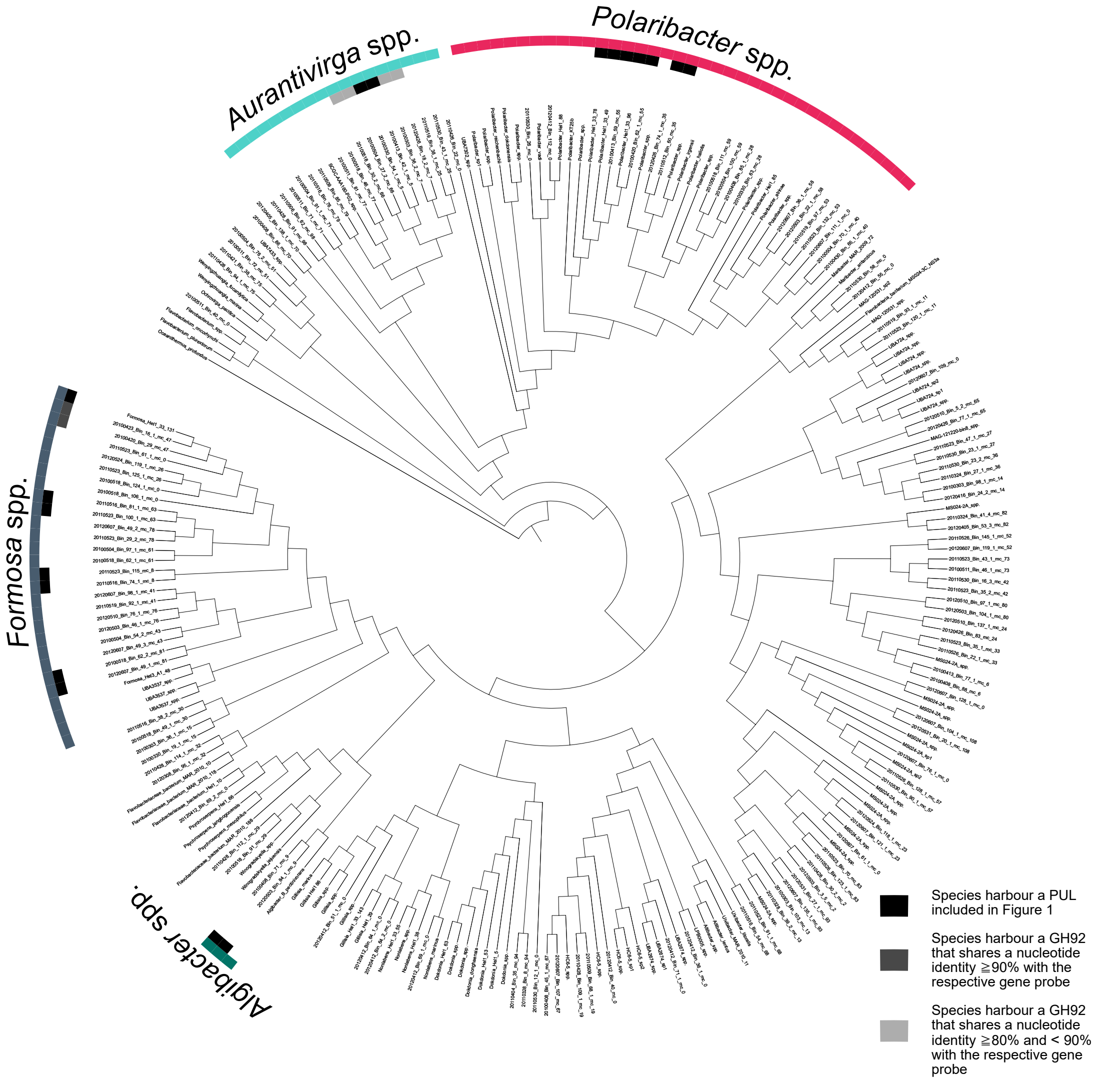

Supplementary Figure 1: Radial phylogenetic tree (reduced version of tree published in (Krüger et al., 2019)) displaying *Flavobacteriaceae*, including the four genera *Formosa*, *Polaribacter*, *Aurantivirga* and *Algibacter*. The shading of the inner circle indicates, which Mash cluster (mc; represented in the tree by two MAGs each) harbours the targeted GH92s either in a PUL displayed in Figure 1 (black), or with at least a nucleotide identity of ≧ 90 (dark grey) or of ≧ 80% and < 90% (light grey). Details for probe hits can be found in Supplementary Table 2.

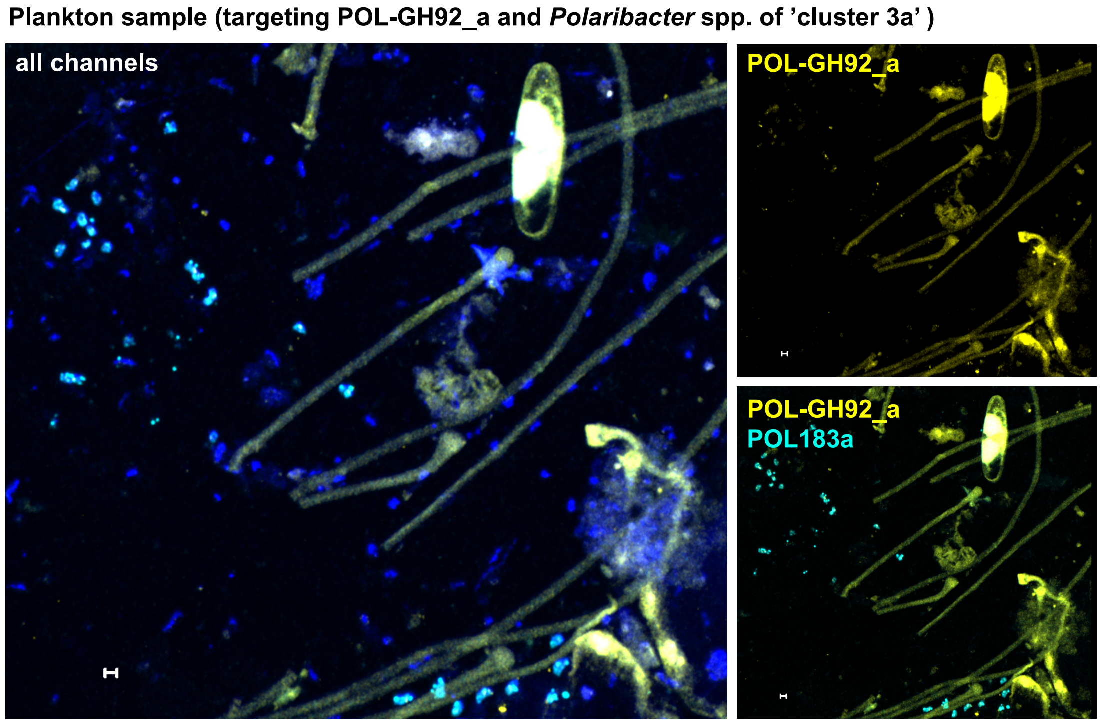

Supplementary Figure 2: Detection of POL-GH92_a genes with direct-geneFISH in plankton samples taken during the spring bloom in the German Bight on 26th April 2010. *Polaribacter* spp. of 'cluster 3a', targeted with the 16S rRNA probe POL183a, are partly attached to algae debris and partly encountered free-living. DNA was counterstained with DAPI (appears blue in 'all channels'). Shown micrographs are maximum intensity projections of processed images achieved with Airyscan microscopy in super-resolution mode. Scale bar: 1 µm.

Helgoland sample (targeting POL-GH92_a and *Polaribacter* spp. of 'cluster 3a')

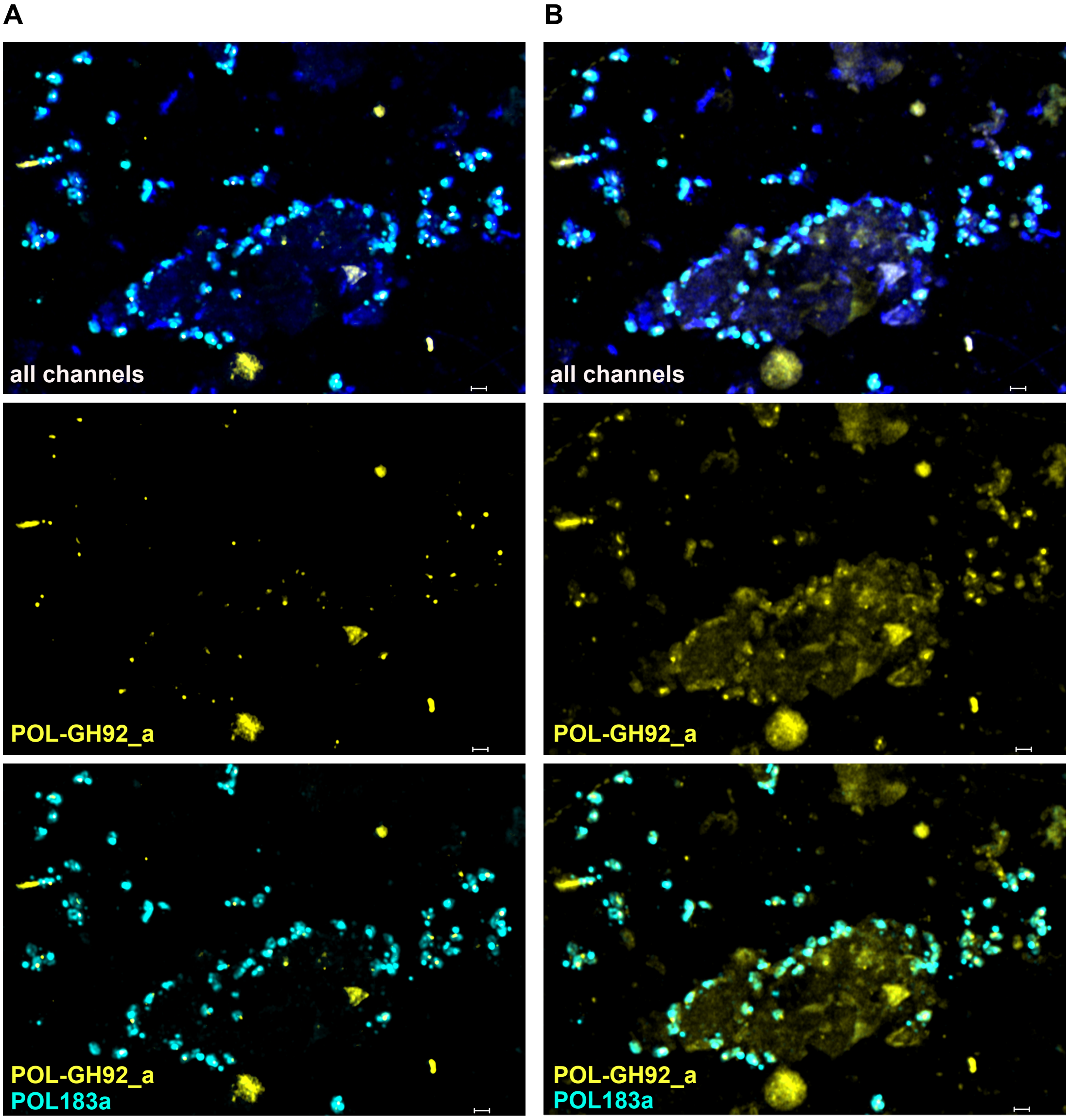

Supplementary Figure 3: Exemplary micrographs of GH92_a detection with POL-GH92_a gene probe set in *Polaribacter* spp. of 'cluster 3a' identified with 16S rRNA probe POL183a with direct-geneFISH in a plankton sample taken on 20th April 2010 in the German Bight. The majority of targeted cells are clearly attached to algae debris. **A** Micrograph showing plankton sample with computationally suppressed autofluorescence. The histogram of intensities for the channel showing the emission between 500-550 nm is set in Zen software to mainly highlight the gene signals. **B** Micrograph showing plankton sample with autofluorescence. Histogram of intensities for the channel showing the emission between 500-550 nm is set in Zen software to display the gene signals and also the autofluorescence of the sample when excited with a 488 nm laser. DNA was counterstained with DAPI (appears blue in 'all channels'). Shown micrographs are maximum intensity projections of processed images achieved with Airyscan microscopy in super-resolution mode. Scale bar: 1 µm.

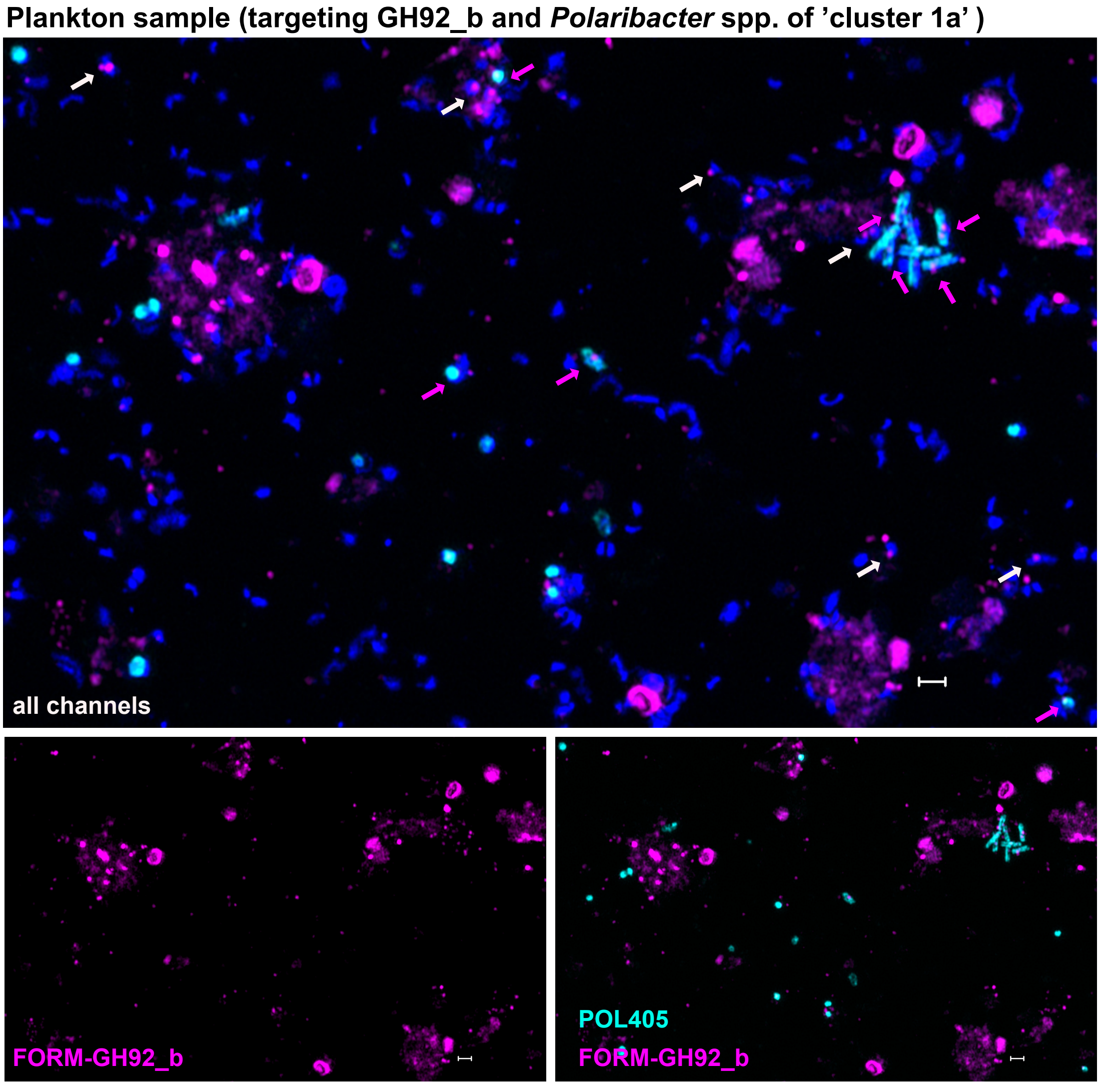

Supplementary Figure 4: Exemplary micrographs of GH92_b detection with FORM-GH92_b gene probe set in *Polaribacter* spp. of 'cluster 1a' identified with 16S rRNA probe POL405 with direct-geneFISH in a plankton sample taken on 3rd May 2012 in the German Bight. The majority of targeted cells are not attached to algae debris. Cells in the upper right corner display a very different morphology than the other targeted cells, hinting towards different species being targeted by the 'cluster 1a' specific 16S rRNA probe. Arrows in magenta show the detected gene signals, white arrows display gene-like signals in other non-target organisms. DNA was counterstained with DAPI (appears blue in 'all channels'). Shown micrographs are maximum intensity projections of processed images achieved with Airyscan microscopy in super-resolution mode. Scale bar: 1 µm.

Formosa Hel1_33_131	FORM-GH92_a_1		FORM-GH92_a_2		FORM-GH92_a_3		FORM-GH92_a_4		FORM-GH92_a_5	
	Pairwise identity [%]	Longest stretch of mismatch/ gap [bp]	Pairwise identity [%]	Longest stretch of mismatch/ gap [bp]	Pairwise identity [%]	Longest stretch of mismatch/ gap [bp]	Pairwise identity [%]	Longest stretch of mismatch/ gap [bp]	Pairwise identity [%]	Longest stretch of mismatch/ gap [bp]
FORM-GH92a	100	-	100	-	100	-	100	-	100	-
NODE_12-GH92 -a	80.0	3	75.8	3	85.5	2	89.1	3	85.3	2
NODE_1292-GH92 -a	81.1	3	75.7	4	86.0	2	86.4	3	85.9	2
NODE_1379-GH92_a	87.7	12	91.4	4	93.6	2	94.0	2	91.2	2
NODE_154-GH92_a	88.1	12	90.3	2	92.3	2	94.0	2	92.5	2
NODE_193-GH92_a	85.7	12	88.8	4	93.2	2	92.9	3	91.6	1
NODE_2497-GH92_a	92.8	2	90.6	2	93.6	2	94.7	2	93.4	1
NODE_3470-GH92_a	96.0	2	86.5	4	90.4	2	91.3	3	90.7	2
POL-GH92_a	80.0	3	75.8	3	85.5	2	89.1	3	85.3	2
S3-860_GH92-a	84.9	3	89.2	2	89.9	2	89.8	3	86.9	2

Formosa Hel1_33_131	FORM-GH92_b_1		FORM-GH92_b_2		FORM-GH92_b_3		FORM-GH92_b_4		FORM-GH92_b_5	
	Pairwise identity [%]	Longest stretch of mismatch/ gap [bp]	Pairwise identity [%]	Longest stretch of mismatch/ gap [bp]	Pairwise identity [%]	Longest stretch of mismatch/ gap [bp]	Pairwise identity [%]	Longest stretch of mismatch/ gap [bp]	Pairwise identity [%]	Longest stretch of mismatch/ gap [bp]
FORM-GH92_b	100	-	100	-	100	-	100	-	100	-
NODE_12-GH92_b	83.3	6	86.3	2	87.3	2	90.5	2	84.1	3
NODE_1292-GH92_b	83.9	3	86.3	2	87.3	2	90.3	2	84.1	3
NODE_1379-GH92_b	84.2	6	91.4	2	87.5	2	90.5	2	84.1	3
NODE_154-GH92_b	87.3	3	89.9	3	93.4	2	92.8	2	90.7	3
NODE_193-GH92_b	87.5	3	92.7	2	92.3	2	93	2	87.4	2
NODE_2277-GH92_b	74.1	4	73.2	6	73.5	4	78.4	4	73.7	4
NODE_2497-GH92_b	92.7	3	90.8	2	93.6	2	93.3	2	92.9	3
NODE_334-GH92_b	81.5	5	85.7	4	85.3	3	87.6	3	80.9	3
NODE_3470-GH92_b	86.8	3	93.0	1	93.8	2	93.9	2	95.8	1
NODE_910-GH92_b	82.2	4	76.4	5	79.8	3	85.8	3	83.0	4
POL-GH92_b	83.3	6	86.3	2	87.3	2	90.5	2	84.1	3
S3-860_GH92-b	85.7	6	90.5	2	89.1	2	92.3	1	87.1	2

Polaribacter Hel1_33_49	POL-GH92_a_1		POL-GH92_a_2		POL-GH92_a_3		POL-GH92_a_4		POL-GH92_a_5	
	Pairwise identity [%]	Longest stretch of mismatch/ gap [bp]	Pairwise identity [%]	Longest stretch of mismatch/ gap [bp]	Pairwise identity [%]	Longest stretch of mismatch/ gap [bp]	Pairwise identity [%]	Longest stretch of mismatch/ gap [bp]	Pairwise identity [%]	Longest stretch of mismatch/ gap [bp]
FORM-GH92_a	80.1	2	76.3	3	85.6	2	89.2	3	85.5	2
NODE_12-GH92_a	100	0	100	0	100	0	100	0	100	0
NODE_1292-GH92_a	97.8	1	98.4	1	96.4	1	93.1	2	87.5	4
NODE_1379-GH92_a	84.9	3	78.5	4	84.7	3	88.5	4	88.2	2
NODE_154-GH92_a	81.3	12	76.5	6	84.9	3	87.8	3	85.7	4
NODE_193-GH92_a	85.2	3	77.4	5	85.4	3	89.0	3	86.6	2
NODE_2497-GH92_a	81.1	3	76.1	5	84.4	3	87.6	4	85.3	2
NODE_3470-GH92_a	80.6	3	75.4	3	83.8	3	87.2	3	85.5	3
POL-GH92_a	100	-	100	-	100	-	100	-	100	-
S3-860_GH92-a	85.9	3	76.1	6	85.6	4	90.3	3	87.9	2

Supplementary Table 1: Pairwise identity in [%] and longest stretch of mismatches in [bp] for each of the 15 probes of the three probe sets FORM-GH92_a_1-5, _b_1-5 and POL-GH92_a_1-5 compared to each of the equivalent GH92-variants from the analysed putative glucuronomannan PULs in Figure 1.

	Total number of gene hits		Hits on contigs not represented in Figure 1		# of taxonomically classified contigs not represented in Figure 1		Taxonomic classification	
	≥80%,<90%	≥90%	≥80%,<90%	≥90%	≥80%,<90%	≥90%	≥80%,<90%	≥90%
**FORM-GH92_a**	16	25	7	10	5	3	mc_9, mc_63, mc_81	mc_8, mc_47
**FORM-GH92_b**	38	18	12	16	7	8	mc_5, mc_7, mc_8, mc_66	mc_35, mc_47, mc_63, mc_64, mc_81
**POL-GH92_a**	7	12	1	6	1	4	mc_81	mc_9, mc_55

**Taxonomic classification**		
	**GTDB r83**	
**mc_5**	SCGC-AAA160-P02	
**mc_7**	SCGC-AAA160-P02	
**mc_8**	UBA3537	
**mc_9**	Algibacter_B	
**mc_35**	Polaribacter	
**mc_47**	UBA3537	
**mc_55**	Polaribacter	
**mc_63**	UBA3537	GCF_001735745.1
**mc_64**	UBA3537	
**mc_66**	SCGC-AAA160-P02	
**mc_81**	UBA3537	

Supplementary Table 2: Taxonomic classification of gene hits that are targeted by our gene probes but are not found in PULs specified in Figure 1. At least four of five probes per set needed to have a blast hit to the same metagenome GH92, with specified percent identity of both $\geq$ 80% and $\leq$ 90 or $\geq$ 90%.

**Probe name**	**Target group**	**Probe sequence (5' → 3')**	**FA [%]**	**Reference**
EUB338-I	*Bacteria*	GCTGCCTCCCGTAGGAGT	35	Amann et al., 1990
EUB338-II	Supplement to EUB338	GCAGCCACCCGTAGGTGT	35	Daims et al., 1999
EUB338-III	Supplement to EUB338	GCTGCCACCCGTAGGTGT	35	Daims et al., 1999
FORM181B	*Formosa* Hel1_33_131	GATGCCACTCTTAGAGAC	35	Teeling et al., 2016
FORM181B-c	Competitor for FORM181B	GATGCCACTCTAAGAGAC	35	Teeling et al., 2016
FORM181B-h1	Helper for FORM181B	CCGTAACCTTTACTCTAAATGT	35	this study
FORM181B-h2	Helper for FORM181B	TATGGGGGGTTAATCTTCATTT	35	this study
POL183a	*Polaribacter* 'cluster 3a'	CTCGATGCCAAGTCTCAA	15	Avcı et al., 2020
POL183a-c5	Competitor for POL183a	AACTTGATGCCAAGTCTCAA	15	Avcı et al., 2020
POL183a-c10	Competitor for POL183a	CTCGATGCCGAGTCTCAATA	15	Avcı et al., 2020
POL183a-c15	Competitor for POL183a	CTCGATGCCAAGTCGCAATA	15	Avcı et al., 2020
POL183a-h1	Helper for POL183a	TACTATAAGGTATTAATCTTCA	15	Avcı et al., 2020
POL183a-h2	Helper for POL183a	TAAATCTTTAATTAAAA	15	Avcı et al., 2020
POL405	*Polaribacter* 'cluster 1a'	CCCATAGGGCATTCTTCCTACA	20	Avcı et al., 2020
POL405-c13	Competitor for POL405	ACCCATAGGGCAGTCTTCCTACA	20	Avcı et al., 2020
POL405-c21	Competitor for POL405	ACCCATAGGGCATTCTTCCTBCA	20	Avcı et al., 2020
POL405-h1	Helper for POL405	CGCRGCATGGCKGGATCAGAGTCTC	20	Avcı et al., 2020
POL405-h2	Helper for POL405	TTCYTCCTGTATAAAAGTAGTTTACAA	20	Avcı et al., 2020

Supplementary Table 3: Specific oligonucleotide probes used in this study to target the 16S rRNA for cell identification. FA: formamide concentration [%] in the hybridization buffer recommended in respective references.

Probes and primers for targeting POL-GH92_a (2253 bp, PHEL49_1329) on *Polaribacter* Hel1_33_49									
				PCR forward primers			PCR reverse primers		
Probe	Region in genome	Length [bp]	GC [%]	Sequence 5' → 3'	Length [bp]	Tm [°C]	Sequence 5' → 3'	Length [bp]	Tm [°C]
POL-GH92_a probe 1	1452329-1452775	447	31	GAATTTAGTATTTAATAAACAAC	23	44	AAAATACCTGCTTGTGC	17	49
POL-GH92_a probe 2	1452776-1453224	449	32	TAGATTCAGTTATCAAAA	18	41	TATTTATCATTCTTTCATAAG	21	43
POL-GH92_a probe 3	1453228-1453674	447	35	CTGAAGATGGAAAATATTATAG	22	47	GCACTCTTGATTCAGTTT	18	50
POL-GH92_a probe 4	1453675-1454122	448	34	ACGGTTTTGAAAAAAGACAATCT	23	54	GGTTCATTTCCCATAGAATA	20	49
POL-GH92_a probe 5	1454123-1454571	449	32	AAGTTTTCATATTCCATA	18	42	TAAAATTGTTTGGAATGA	18	42

Probes and primers for targeting FORM-GH92_a (2256 bp, FORMB_02890) and FORM-GH92_b (2280 bp, FORMB_02900) on *Formosa* Hel1_33_131									
				PCR forward primers			PCR reverse primers		
Probe	Region in genome	Length [bp]	GC [%]	Sequence 5' → 3'	Length [bp]	Tm [°C]	Sequence 5' → 3'	Length [bp]	Tm [°C]
FORM-GH92_a probe 1	312869-313314	446	35	TGAATTTAAAGACCTTTATA	20	42	TGATTGCGCTGCGGGTGT	18	61
FORM-GH92_a probe 2	313317-313763	447	34	TTTCTTTAAATTTACCT	17	37	ATCATTCGTTCATAGGAA	18	46
FORM-GH92_a probe 3	313764-314219	456	40	AAATATTTCTGAAGACGG	18	45	TGAACTCTAGGCTCTGTTT	19	51
FORM-GH92_a probe 4	314221-314670	450	35	GATTTTGAAAAAAGAC	16	38	ACTGGGTTCATTTCCCATA	19	51
FORM-GH92_a probe 5	314671-315112	442	38	TTTCATATCCCCTATT	16	40	AATCGTTTGGTATCAG	19	43
FORM-GH92_b probe 1	315137-315591	455	40	ATGAAAAATATAAAAATCTAC	21	41	TTGATGTTGTAATCTTTTAG	20	46
FORM-GH92_b probe 2	315592-316046	455	33	AGCAGAACTGACGGCTTCAA	20	58	CTTTTTTCCAAGTTTCATGC	20	50
FORM-GH92_b probe 3	316063-316517	455	36	TGTCAAAGGAGGTACTGA	18	47	TTTTAACTCTTTCTGATGGA	20	47
FORM-GH92_b probe 4	316518-316963	446	35	ATATTTTAATGCCTAAGGGA	20	47	ATCTCCAGAAACATCATCT	19	50
FORM-GH92_b probe 5	316964-317416	453	36	ATTTCGGGTCTCATTGGG	18	54	TTACTTTGGTTGCTTACCAC	20	51

Supplementary Table 5: Polynucleotide probes and respective primer design for all three probe sets targeting GH92s in this study (see also Supplementary Data 2 for full polynucleotide gene sequences).

Probe set targeting FORM- GH92_a and _b			
Probe	Label	DOL	Total # of dyes
FORM-GH92_a probe 1	Alexa 594	3.7	77
FORM-GH92_a probe 2		0.5	
FORM-GH92_a probe 3		4.0	
FORM-GH92_a probe 4		4.1	
FORM-GH92_a probe 5		4.9	
FORM-GH92_b probe 1	Alexa 647	2.7	69
FORM-GH92_b probe 2		2.0	
FORM-GH92_b probe 3		4.3	
FORM-GH92_b probe 4		4.3	
FORM-GH92_b probe 5		1.9	
Probe mix FORM-GH92_a	A647	1.3	29
Probe mix FORM-GH92_b	A594	2.8	63

Probe set targeting POL- GH92_a			
Probe	Label	DOL	Total # of dyes
POL-GH92_a probe 1	Alexa 488	1.8	38
POL-GH92_a probe 2		1.6	
POL-GH92_a probe 3		2.2	
POL-GH92_a probe 4		2.2	
POL-GH92_a probe 5		0.7	

Supplementary Table 6: The amount of dyes per probe sets and the degree of labelling (DOL) for each probe, calculated as the amount of dye molecules per 100 bp of polynucleotide probe.

	Airyscan	
Fluorophore	Excitation laser [nm]	Detection window [nm]
DAPI	405	420-480
Atto488	488	500-550
Cy3	561	570-615
Alexa594	561	570-615
Alexa647	633	LP 655

Supplementary Table 7: Settings for Airyscan microscopy. LP = long pass, nm = nanometre.

	FORM-GH92_a	NODE_2497-GH92_a	NODE_154-GH92_a	NODE_1379-GH92_a	NODE_3470-GH92_a	NODE_193-GH92_a	S3-860-GH92_a	NODE_12-GH92_a	POL-GH92_a	NODE_1292-GH92_a	FORM-GH92_c	NODE_154-GH92_c	NODE_193-GH92_c	S3-860-GH92_c	POL-GH92_c	NODE_334-GH92_c	NODE_910-GH92_c	NODE_2277-GH92_c	FORM-GH92_b	NODE_2497-GH92_b	NODE_193-GH92_b	S3-860-GH92_b	NODE_3470-GH92_b	NODE_154-GH92_b	NODE_12-GH92_b	POL-GH92_b	NODE_1379-GH92_b	NODE_1292-GH92_b	NODE_334-GH92_b	NODE_910-GH92_b	NODE_2277-GH92_b	POL-GH92_d	FORM-GH92_d	NODE_154-GH92_d	POL-GH92_e	NODE_334-GH92_d	NODE_910-GH92_d
FORM-GH92_a		93.0	91.5	91.5	91.0	90.4	87.7	82.7	82.7	81.9	57.1	57.0	56.9	57.9	56.8	57.6	57.3	55.0	45.4	44.9	45.4	46.0	43.9	46.0	46.2	46.2	45.9	46.2	45.8	46.1	45.0	41.9	43.4	43.5	43.0	43.3	43.0
NODE_2497_GH92-a	93.0		92.1	91.1	90.0	89.5	87.1	82.2	82.2	81.0	56.6	56.6	56.4	57.1	56.1	57.8	57.6	54.9	45.0	44.5	45.3	46.0	43.5	45.7	46.1	46.1	46.0	45.9	45.4	45.6	44.7	41.4	42.8	42.7	42.2	43.4	43.2
NODE_154_GH92-a	91.5	92.1		91.7	88.7	90.6	87.9	82.8	82.8	81.7	57.7	57.5	57.5	58.0	57.3	57.5	57.2	55.9	45.2	44.6	45.1	45.9	44.1	45.8	45.8	45.8	45.7	46.0	45.3	45.2	44.4	41.4	42.8	43.0	42.7	42.9	42.8
NODE_1379_GH92-a	91.5	91.1	91.7		88.7	90.8	87.8	83.4	83.4	81.8	57.5	57.3	57.8	58.2	57.2	57.7	57.5	55.2	45.0	44.6	45.7	46.1	44.1	45.9	46.0	46.0	45.7	46.5	45.6	45.7	44.8	41.8	43.3	43.4	43.5	43.8	43.4
NODE_3470_GH92-a	91.0	90.0	88.7	88.7		89.6	86.4	82.1	82.1	81.0	56.6	56.6	56.7	57.4	56.2	57.0	56.8	55.3	45.2	44.9	45.1	45.6	43.7	45.2	45.5	45.5	45.6	45.2	44.9	44.8	44.3	41.9	42.7	43.0	42.4	43.2	43.1
NODE_193_GH92-a	90.4	89.5	90.6	90.8	89.6		88.8	82.9	82.9	81.5	56.9	57.0	57.4	57.7	57.1	57.4	56.6	55.1	45.6	45.0	45.9	46.3	44.2	46.0	46.3	46.3	46.3	46.3	45.6	45.3	44.4	41.8	42.8	43.2	43.0	43.5	43.7
S3-860-GH92_a	87.7	87.1	87.9	87.8	86.4	88.8		85.0	85.0	83.9	57.6	57.9	57.8	58.5	58.0	58.0	57.7	55.8	46.1	45.7	46.1	47.1	44.8	46.7	47.0	47.0	47.1	46.6	45.5	45.8	44.7	42.8	43.9	43.6	44.0	43.7	44.4
NODE_12_GH92-a	82.7	82.2	82.8	83.4	82.1	82.9	85.0		100.0	94.1	58.2	58.7	58.1	58.5	57.8	59.3	59.0	56.7	46.5	45.8	46.6	46.9	44.5	46.7	47.8	47.8	47.9	47.1	46.4	46.5	45.0	43.1	44.3	44.4	44.6	44.3	44.7
POL-GH92_a	82.7	82.2	82.8	83.4	82.1	82.9	85.0	100.0		94.1	58.2	58.7	58.1	58.5	57.8	59.3	59.0	56.7	46.5	45.8	46.6	46.9	44.5	46.7	47.8	47.8	47.9	47.1	46.4	46.5	45.0	43.1	44.3	44.4	44.6	44.3	44.7
NODE_1292-GH92_a	81.9	81.0	81.7	81.8	81.0	81.5	83.9	94.1	94.1		57.8	57.8	57.6	57.7	56.8	59.0	58.6	56.6	45.8	45.6	46.5	46.8	44.3	47.0	47.3	47.3	47.5	46.7	46.7	46.5	45.0	42.3	44.3	44.4	44.4	44.6	44.9
FORM-GH92_c	57.1	56.6	57.7	57.5	56.6	56.9	57.6	58.2	58.2	57.8		93.1	90.7	92.8	91.7	75.5	76.6	71.6	45.6	45.0	45.7	45.6	43.7	45.2	45.4	45.4	45.5	45.4	45.0	45.5	44.2	41.2	40.9	40.8	40.9	40.0	39.6
NODE_154-GH92_c	57.0	56.6	57.5	57.3	56.6	57.0	57.9	58.7	58.7	57.8	93.1		90.9	91.2	91.3	75.9	76.3	72.4	45.4	44.8	45.5	45.5	44.0	45.2	46.2	46.2	46.2	45.8	45.4	45.6	44.8	41.5	41.0	40.9	40.6	40.0	39.9
NODE_193-GH92_c	56.9	56.4	57.5	57.8	56.7	57.4	57.8	58.1	58.1	57.6	90.7	90.9		93.0	90.5	76.4	75.9	72.2	45.7	45.2	46.2	46.0	44.1	45.6	46.3	46.3	46.5	46.1	45.3	45.8	44.1	41.8	40.5	40.6	40.5	40.6	40.1
S3-860-GH92_c	57.9	57.1	58.0	58.2	57.4	57.7	58.5	58.5	58.5	57.7	92.8	91.2	93.0		91.4	76.9	77.3	72.2	45.8	45.2	46.1	46.1	44.2	45.7	46.3	46.3	46.5	46.2	45.4	45.8	44.3	41.6	41.2	41.0	41.1	40.8	40.4
Pol-GH92_c	56.8	56.1	57.3	57.2	56.2	57.1	58.0	57.8	57.8	56.8	91.7	91.3	90.5	91.4		76.1	76.2	71.8	44.9	44.6	45.1	45.2	43.7	45.0	46.0	46.0	45.9	45.7	44.8	45.6	43.7	40.8	40.9	40.5	41.1	40.3	40.0
NODE_334-GH92_c	57.6	57.8	57.5	57.7	57.0	57.4	58.0	59.3	59.3	59.0	75.5	75.9	76.4	76.9	76.1		93.9	73.6	44.4	44.3	44.7	44.9	43.2	44.2	45.7	45.7	45.8	45.3	44.7	44.7	42.7	40.9	41.0	41.0	40.7	41.3	40.5
NODE_910-GH92_c	57.3	57.6	57.2	57.5	56.8	56.6	57.7	59.0	59.0	58.6	76.6	76.3	75.9	77.3	76.2	93.9		73.7	44.0	43.5	44.4	44.8	43.3	44.0	45.6	45.6	45.7	45.1	44.4	44.2	43.1	40.9	40.7	40.5	40.0	41.3	40.8
NODE_2277-GH92_c	55.0	54.9	55.9	55.2	55.3	55.1	55.8	56.7	56.7	56.6	71.6	72.4	72.2	72.2	71.8	73.6	73.7		42.9	42.6	42.8	43.2	41.0	43.0	44.2	44.2	44.2	44.5	43.7	43.3	43.0	40.0	40.8	40.6	39.7	40.9	41.4
FORM-GH92_b	45.4	45.0	45.2	45.0	45.2	45.6	46.1	46.5	46.5	45.8	45.6	45.4	45.7	45.8	44.9	44.4	44.0	42.9		92.6	90.3	88.6	92.2	90.8	86.0	86.0	87.3	84.6	82.7	79.0	71.7	54.2	46.1	46.3	46.5	46.4	46.0
NODE_2497-GH92_b	44.9	44.5	44.6	44.6	44.9	45.0	45.7	45.8	45.8	45.6	45.0	44.8	45.2	45.2	44.6	44.3	43.5	42.6	92.6		89.2	88.2	90.2	91.0	85.6	85.6	86.9	84.5	82.7	78.7	72.2	54.0	46.1	46.5	46.7	46.5	46.3
NODE_193-GH92_b	45.4	45.3	45.1	45.7	45.1	45.9	46.1	46.6	46.6	46.5	45.7	45.5	46.2	46.1	45.1	44.7	44.4	42.8	90.3	89.2		93.8	92.1	91.1	87.7	87.7	89.1	86.8	82.7	79.6	73.3	54.5	46.7	47.2	47.4	47.6	46.7
S3-860-GH92_b	46.0	46.0	45.9	46.1	45.6	46.3	47.1	46.9	46.9	46.8	45.6	45.5	46.0	46.1	45.2	44.9	44.8	43.2	88.6	88.2	93.8		89.5	90.4	89.1	89.1	90.5	87.4	83.7	80.0	73.6	55.0	46.7	46.8	47.4	47.2	46.7
NODE_3470-GH92_b	43.9	43.5	44.1	44.1	43.7	44.2	44.8	44.5	44.5	44.3	43.7	44.0	44.1	44.2	43.7	43.2	43.3	41.0	92.2	90.2	92.1	89.5		91.1	87.7	87.7	88.2	86.4	82.9	78.3	73.3	53.8	45.8	46.3	46.6	46.3	45.7
NODE_154-GH92_b	46.0	45.7	45.8	45.9	45.2	46.0	46.7	46.7	46.7	47.0	45.2	45.2	45.6	45.7	45.0	44.2	44.0	43.0	90.8	91.0	91.1	90.4	91.1		87.0	87.0	88.0	86.2	83.2	79.5	72.1	54.4	46.4	46.5	46.8	47.2	46.6
NODE_12-GH92_b	46.2	46.1	45.8	46.0	45.5	46.3	47.0	47.8	47.8	47.3	45.4	46.2	46.3	46.3	46.0	45.7	45.6	44.2	86.0	85.6	87.7	89.1	87.7	87.0		100.0	97.2	96.2	87.6	81.0	73.1	55.1	47.5	47.8	48.3	47.6	47.0
POL-GH92_b	46.2	46.1	45.8	46.0	45.5	46.3	47.0	47.8	47.8	47.3	45.4	46.2	46.3	46.3	46.0	45.7	45.6	44.2	86.0	85.6	87.7	89.1	87.7	87.0	100.0		97.2	96.2	87.6	81.0	73.1	55.1	47.5	47.8	48.3	47.6	47.0
NODE_1379-GH92_b	45.9	46.0	45.7	45.7	45.6	46.3	47.1	47.9	47.9	47.5	45.5	46.2	46.5	46.5	45.9	45.8	45.7	44.2	87.3	86.9	89.1	90.5	88.2	88.0	97.2	97.2		93.6	85.4	80.6	73.3	55.3	47.6	47.4	48.0	47.4	46.9
NODE_1292-GH92_b	46.2	45.9	46.0	46.5	45.2	46.3	46.6	47.1	47.1	46.7	45.4	45.8	46.1	46.2	45.7	45.3	45.1	44.5	84.6	84.5	86.8	87.4	86.4	86.2	96.2	96.2	93.6		87.2	80.5	72.8	55.2	47.5	47.7	48.2	47.8	47.1
NODE_334-GH92_b	45.8	45.4	45.3	45.6	44.9	45.6	45.5	46.4	46.4	46.7	45.0	45.4	45.3	45.4	44.8	44.7	44.4	43.7	82.7	82.7	82.7	83.7	82.9	83.2	87.6	87.6	85.4	87.2		87.1	72.8	55.5	45.4	45.9	46.4	46.5	46.0
NODE_910-GH92_b	46.1	45.6	45.2	45.7	44.8	45.3	45.8	46.5	46.5	46.5	45.5	45.6	45.8	45.8	45.6	44.7	44.2	43.3	79.0	78.7	79.6	80.0	78.3	79.5	81.0	81.0	80.6	80.5	87.1		72.8	54.4	44.6	45.1	46.0	45.4	45.2
NODE_2277-GH92_b	45.0	44.7	44.4	44.8	44.3	44.4	44.7	45.0	45.0	45.0	44.2	44.8	44.1	44.3	43.7	42.7	43.1	43.0	71.7	72.2	73.3	73.6	73.3	72.1	73.1	73.1	73.3	72.8	72.8	72.8		52.3	45.6	46.1	45.7	45.9	45.8
POL-GH92_d	41.9	41.4	41.4	41.8	41.9	41.8	42.8	43.1	43.1	42.3	41.2	41.5	41.8	41.6	40.8	40.9	40.9	40.0	54.2	54.0	54.5	55.0	53.8	54.4	55.1	55.1	55.3	55.2	55.5	54.4	52.3		44.9	44.3	45.1	44.9	45.0
FORM-GH92_d	43.4	42.8	42.8	43.3	42.7	42.8	43.9	44.3	44.3	44.3	40.9	41.0	40.5	41.2	40.9	41.0	40.7	40.8	46.1	46.1	46.7	46.7	45.8	46.4	47.5	47.5	47.6	47.5	45.4	44.6	45.6	44.9		93.2	89.2	78.1	77.6
NODE_154-GH92_d	43.5	42.7	43.0	43.4	43.0	43.2	43.6	44.4	44.4	44.4	40.8	40.9	40.6	41.0	40.5	41.0	40.5	40.6	46.3	46.5	47.2	46.8	46.3	46.5	47.8	47.8	47.4	47.7	45.9	45.1	46.1	44.3	93.2		88.5	77.5	77.2
POL-GH92_e	43.0	42.2	42.7	43.5	42.4	43.0	44.0	44.6	44.6	44.4	40.9	40.6	40.5	41.1	41.1	40.7	40.0	39.7	46.5	46.7	47.4	47.4	46.6	46.8	48.3	48.3	48.0	48.2	46.4	46.0	45.7	45.1	89.2	88.5		79.4	79.3
NODE_334-GH92_d	43.3	43.4	42.9	43.8	43.2	43.5	43.7	44.3	44.3	44.6	40.0	40.0	40.6	40.8	40.3	41.3	41.3	40.9	46.4	46.5	47.6	47.2	46.3	47.2	47.6	47.6	47.4	47.8	46.5	45.4	45.9	44.9	78.1	77.5	79.4		88.7
NODE_910-GH92_d	43.0	43.2	42.8	43.4	43.1	43.7	44.4	44.7	44.7	44.9	39.6	39.9	40.1	40.4	40.0	40.5	40.8	41.4	46.0	46.3	46.7	46.7	45.7	46.6	47.0	47.0	46.9	47.1	46.0	45.2	45.8	45.0	77.6	77.2	79.3	88.7	

Supplementary Table 8: Percentage identities based on the nucleic acid sequences of all GH92 genes in putative glucuronomannan PULs from 38 metagenomes obtained from spring bloom sampling campaigns in the North Sea between 2010-2012. For better understanding, FORM-GH92_a, _b and POL-GH92_a, for which polynucleotide probes were designed, are highlighted by framed rows. Light grey indicates percentage identities ≧ 80% and dark grey ≧ 90%. Empty fields indicate 100% identity.

	FA [%]	TFm [°C]	Tmin [°C]	Tmax [°C]	Mismatches [%]
**FORM_GH92_b_1**	15	70	40	50	20
	35	67	37	47	10
**FORM_GH92_b_2**	15	67	37	47	20
	35	69	39	49	5
**FORM_GH92_b_3**	15	68	38	48	20
	35	66	36	46	10
**FORM_GH92_b_4**	15	68	38	48	20
	35	70	40	50	5
**FORM_GH92_b_5**	15	68	38	48	20
	35	66	36	46	10

Supplementary Table 9: Percentage of mismatches calculated for relaxed (15% FA) and stringent (35% FA) hybridization conditions of polynucleotide probes FORM-GH92_b_1-5. TFm = melting temperature corrected for the used FA concentration, FA = formamide in hybridization buffer.
